# Direct whole-genome sequencing of *Plasmodium falciparum* specimens from dried erythrocyte spots

**DOI:** 10.1186/s12936-018-2232-6

**Published:** 2018-02-23

**Authors:** Sidsel Nag, Poul-Erik Kofoed, Johan Ursing, Camilla Koldbæk Lemvigh, Rosa Lundbye Allesøe, Amabelia Rodrigues, Christina Aaby Svendsen, Jacob Dyring Jensen, Michael Alifrangis, Ole Lund, Frank M. Aarestrup

**Affiliations:** 1 Centre for Medical Parasitology at Department of Immunology and Microbiology, Faculty of Health and Medical Science, University of Copenhagen, Copenhagen, Denmark; 2 Department of Infectious Diseases, Copenhagen University Hospital (Rigshospitalet), Copenhagen, Denmark; 3 Department of Paediatrics, Kolding Hospital, Kolding, Denmark; 4 Bandim Health Project, Bissau, Guinea-Bissau; 5 Department of Microbiology, Tumor and Cell Biology, Karolinska Institutet, Stockholm, Sweden; 6 Department of Infectious Diseases, Danderyds Hospital, Danderyd, Sweden; 7 DTU Bioinformatics, Technical University of Denmark, Lyngby, Denmark; 8 Division for Epidemiology and Microbial Genomics, National Food Institute, Technical University of Denmark, Kongens Lyngby, Denmark

**Keywords:** Dried blood spots, Dried erythrocyte spots, Leukocyte depletion, Malaria, *Plasmodium falciparum*, Sub-Saharan Africa, Whole-genome sequencing

## Abstract

**Background:**

*Plasmodium falciparum* malaria remains a major health burden and genomic research represents one of the necessary approaches for continued progress towards malaria control and elimination. Sample acquisition for this purpose is troublesome, with the majority of malaria-infected individuals living in rural areas, away from main infrastructure and the electrical grid. The aim of this study was to describe a low-tech procedure to sample *P. falciparum* specimens for direct whole genome sequencing (WGS), without use of electricity and cold-chain.

**Methods:**

Venous blood samples were collected from malaria patients in Bandim, Guinea-Bissau and leukocyte-depleted using Plasmodipur filters, the enriched parasite sample was spotted on Whatman paper and dried. The samples were stored at ambient temperatures and subsequently used for DNA-extraction. Ratios of parasite:human content of the extracted DNA was assessed by qPCR, and five samples with varying parasitaemia, were sequenced. Sequencing data were used to analyse the sample content, as well as sample coverage and depth as compared to the 3d7 reference genome.

**Results:**

qPCR revealed that 73% of the 199 samples were applicable for WGS, as defined by a minimum ratio of parasite:human DNA of 2:1. WGS revealed an even distribution of sequence data across the 3d7 reference genome, regardless of parasitaemia. The acquired read depths varied from 16 to 99×, and coverage varied from 87.5 to 98.9% of the 3d7 reference genome. SNP-analysis of six genes, for which amplicon sequencing has been performed previously, confirmed the reliability of the WGS-data.

**Conclusion:**

This study describes a simple filter paper based protocol for sampling *P. falciparum* from malaria patients for subsequent direct WGS, enabling acquisition of samples in remote settings with no access to electricity.

**Electronic supplementary material:**

The online version of this article (10.1186/s12936-018-2232-6) contains supplementary material, which is available to authorized users.

## Background

Since the millennium, global efforts towards malaria control and elimination have played a major role in accomplishing an estimated 20% decrease in malaria cases world-wide [1]. As communities proceed towards better control and possible elimination of this disease, it is paramount that the continuous genetic evolution of the malaria parasite populations be investigated [2, 3]. Technological advancements now allow scientists to genetically monitor the parasites and thereby discover genetic adaptations as they occur [4–7]. These analyses are performed through whole-genome sequencing (WGS), a procedure that has become feasible and affordable thanks to next-generation sequencing (NGS) technologies and protocols that circumvent the inherent obstacles pertaining to library preparation of Plasmodium species [8, 9]. The principle obstacle to performing direct WGS of Plasmodium species is the minute quantity of parasite DNA compared with the human DNA in clinical blood samples [8, 9]. This is circumvented by isolating the erythrocytes prior to DNA-extraction (also called leukocyte-depletion). The blood-stage parasites are harboured within the erythrocytes, which in turn do not contain nuclei of their own. Therefore, the DNA extracted primarily belongs to the parasites. Unfortunately, these protocols require electrical equipment and cold-chains for storage, hindering the collection of malaria parasites from rural areas and “hard-to-reach” populations. However, these populations represent the majority of the malaria infections world-wide [3], arguing strongly for their representation in genomic research of *Plasmodium falciparum* specimens. Alternatively, by pre-processing the samples, involving for example selective amplification of the parasite genome (sWGA) [10–14], WGS becomes possible from samples that have not been leukocyte depleted, such as finger-prick samples. Unfortunately, such protocols constrict down-stream analyses to the genomic regions that are effectively amplified [11] while direct WGS would minimize sequencing bias, and allow for more down-stream analyses.

While pre-processing of the samples is unavoidable for low-parasitaemia samples or archival samples that have not been leukocyte-depleted, direct WGS may still be a possibility for infections in remote areas with limited resources, if the sampling procedure is adapted accordingly.

This study describes a simple field applicable protocol for sampling of *P. falciparum* specimens from malaria patients for direct WGS. By directly precipitating and filtering venous blood samples to obtain leukocyte-depleted samples and then collecting these as dried erythrocyte spots (DESs), samples could be processed without electricity and stored without cold-chain, and were later used for direct WGS of the infecting *P. falciparum* specimens. This study provides evidence that the quality of the sequencing data acquired are adequate for further application in genomic research of *P. falciparum.*

## Methods

### Patients and sample collection

Blood samples (N = 199) were collected in Bandim, Guinea-Bissau, which represents many general obstacles encountered when setting up patient sampling in sub-Saharan Africa: the infrastructure is poor, and the connection to the electrical grid is unstable and expensive or completely lacking. The samples were collected from patients with uncomplicated malaria from October 2014 to October 2016. Inclusion criteria were: Informed consent, axillary temperature above 37.5 °C or a history of fever within the previous 24 h. *Plasmodium falciparum* mono-infection, parasite density ≥ 1000 *P. falciparum*/µl, age ≥ 6 months and absence of signs of severe malaria infection. Giemsa-stained thick and thin films were prepared and malaria species identified using a microscope. Parasite densities were calculated by counting the number of *P. falciparum* per 200 white blood cells, or up to 500 parasites. Approximately 2–3 ml of venous blood was drawn from each patient in EDTA-containing vacuum tubes.

**Fig. 1:**
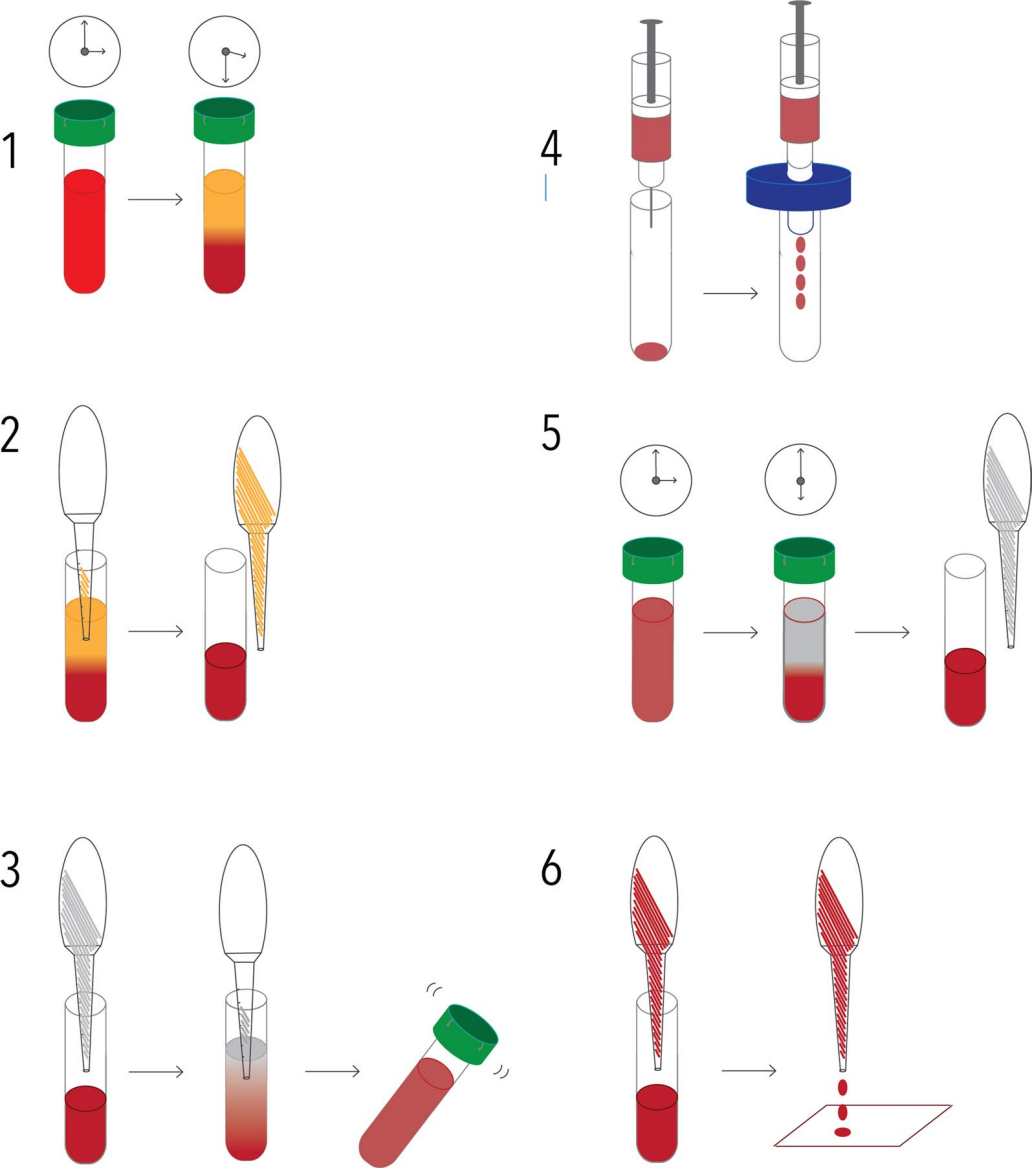
Sampling diagram. Malaria patients donated 2–3 ml of venous blood, which was left to precipitate for approximately 30 min (1) prior to removal of the plasma and buffy coat, using a Pasteur pipette (2). A new Pasteur pipette was used to add PBS to the erythrocytes, and the tube was inverted 3–4 times to mix PBS and erythrocytes (3). The PBS-diluted erythrocytes were then sucked into a syringe, which was applied to a Plasmodipur filter, and pressure was applied until the entire sample had been filtered (4). The filtered PBS-diluted erythrocytes were then left to precipitate for approximately 3 h, before the PBS was removed using yet another Pasteur pipette (5). The erythrocytes were finally dotted on Whatman filter paper #3, as three Pasteur-pipette drops per spot (6)

Leukocyte depletion and dried erythrocyte spots (depicted in Fig. 1).

Venous blood samples were left to precipitate on the counter. Plasma and buffy coat were removed when the erythrocytes had precipitated using a Pasteur pipette. PBS was added to the remaining RBCs, creating approximately a 1:1 dilution and inverted 3–4 times. The RBC fraction + PBS mixture was sucked up using a sterile syringe and needle, and the syringe was then applied to the Plasmodipur filter (Europroxima, Arnhem, The Netherlands, Cat. 8011Filter25U). The mixture was filtered according to manufacturer’s protocol. The filtered mixture was left standing at room temperature for 3 h, to let the erythrocytes precipitate from the PBS. The PBS was carefully removed using a Pasteur pipette, and the erythrocytes were spotted on Whatman paper #3, as 3 Pasteur pipette-drops per spot, and left to dry overnight in closed drawers. Finally, the blood spots were packed in individual zip-lock bags containing desiccant, as well as sample ID numbers. Samples were stored at room temperature (approximately 25–30 °C) in a dark box, for 2–4 months, before shipment to Copenhagen, Denmark. In Copenhagen, the samples were kept at − 20 °C, for 6–8 months before DNA extraction.

### DNA-extraction

DNA was extracted using the QIAamp DNA Mini Kit (Qiagen, Limburg, Netherlands, Cat. 51306), according to WWARN procedures [15], but eluting in 35 μl elution buffer.

### Quantitative PCR

A previously described qPCR method [16] comparing the presence of *P. falciparum* seryl-tRNA synthetase to Human Beta-2-microglobulin, correcting for the size difference between the *P. falciparum* and human genomes was done.

### Library preparation and Miseq sequencing

DNA concentrations in extracts were measured on the Qubit double-stranded DNA (dsDNA) HS assay kit (Invitrogen). Libraries for paired-end sequencing were constructed from DNA extracts ranging from < 50 ng/ml to 0.2 ng/µl, using the Illumina NexteraXT (Illumina, California, USA) Guide 150319425031942 and following protocol revision E. The Pooled NexteraXT libraries were loaded onto an Illumina MiSeq reagent cartridge using MiSeq reagent kit v3 and 500 cycles with a standard flow cell.

### Filtering malaria reads

Paired end reads were analysed with MGmapper v. 2.0 [17, 18], available from the Center for Genomic Epidemiology (CGE). Reads were trimmed from bases with a quality score below 30 (Phred), and paired reads were mapped to the following libraries: (1) malaria, (2) protozoa, (3) bacteria, (4) viruses, (5) fungi and (6) humans, in “bestmode”. The malaria database consists of the 3D7 reference genome, 21 other accessible Plasmodium genomes from NCBI, and contigs generated from *P. falciparum* specimens obtained from malaria patients in Tanzania. The protozoa database does not contain Plasmodium species.

### Alignment and SNP-calling

Alignment of malaria reads to the 3d7 genome was performed using BWA [19], which was then sorted and piled with SAMtools [20], using the–aa option for “absolutely all” bases, to ascertain complete listing of positions with 0 reads. SNP calling was performed with Assimpler as described previously [21].

### Circos imaging

Genomic position information and read depth was cut from the pileup file generated with SAMtools, to generate a separate coverage file, from which average read depths across 2000 bp were calculated. The result was used to visualize read distribution using the Circos software [22].

## Results and discussion

### Sampling protocol overview, implementation and future changes

Figure 1 illustrates the sampling protocol applied, which was developed in order to allow sampling for direct WGS *of P. falciparum* specimens collected in Bandim, Guinea-Bissau, where electricity is scarce, unstable and expensive. The sampling procedure is described in detail in the methods section. Local laboratory assistants were shown once how to perform the procedure, and were equipped with Plasmodipur filters, Pasteur pipettes, falcon tubes, PBS-tablets, Whatman filter paper #3, desiccant, zip lock bags and both a written- and video-presented protocol. The cost of sampling was highly affected by the use of Plasmodipur filters for leukocyte depletion, which were chosen due to the unavailability of the cheaper alternative, CF11 cellulose. An alternative cellulose-product has since been identified [23], which can be used according to the CF11 cellulose protocols and, therefore, represents an inexpensive, yet efficient alternative to Plasmodipur filters [9, 23] for future implementation of this sampling procedure.

### qPCR assessment of human:parasite DNA content

qPCR was performed on all 199 samples to analyse the ratios of parasite:human DNA. This was done in order to assess the applicability of the samples for direct WGS, as defined by a minimum ratio of parasite:human DNA of 2:1. It was decided that samples with human DNA content above this ratio would require excessive sequencing resulting in excessive costs. Samples were categorised as “applicable” or “inapplicable”, according to the threshold. qPCR analysis revealed that 73% (N = 145) of the samples were applicable for WGS. Parasitaemia for the 199 samples varied from 800 parasites/µl to > 81,633 parasites/µl, and logistic regression was performed to establish whether increasing parasitaemia would increase the odds of a sample being applicable for WGS, the input data are listed in Table 1. The acquired odds ratio (OR = 1.29) confirms that, the likelihood of the sample being applicable for direct WGS increases with increasing parasitaemia. The contamination risk is inherently higher when applying this protocol, as it is not performed under sterile conditions. It was therefore assumed that lower-parasitaemia samples would be more difficult to sample successfully, which is the reason for an inclusion criteria of minimum parasitaemia of 1000 parasites/μl. Contamination may also affect higher parasitaemia infections, and the risks include lysis of leukocytes prior to filtration (if for example the blood sample was left for longer than indicated at ambient temperatures), applying too much force during filtration and contamination by anyone handling the samples prior to DNA-extraction.

**Table 1:** Correlation between parasitaemia and sample applicability for direct WGS

Parasitaemia	Applicable count	Applicable %	Inapplicable count	Inapplicable %
< 10,000	28	58	20	42
10,000	48	72	19	28
20,000	5	100	0	0
30,000	20	74	7	26
40,000	24	77	7	23
> 50,000	19	90	2	10

OR = 1.29 (95% CI 1.07–1.58) p = 0.009

Correlation between parasitaemia and sample applicability for direct WGS N = 199, parasitaemias are given as parasites/µl, calculated according to a leukocyte count of 8000 per µl whole blood. Parasites and leukocytes were counted by microscopy, counting until 500 parasites or 200 leukocytes. Samples were grouped in five groups according to parasitaemia, corresponding to intervals of 10,000 parasites/µl. The minimum parasitaemia recorded in group 1 was 800 parasites/µl, and the maximum parasitaemia recorded in group 5 was 81,633 parasites/µl. Applicable/inapplicable count corresponds to the number of samples. Logistic regression was performed to investigate the relationship between parasitaemia of the infection and applicability of the sample for WGS. OR (odds ratio), CI (confidence interval) and p*I*-value (p) are given

### *Plasmodium falciparum* content, coverage and read depth

For the current study, five samples of varying parasitaemia (0.1–1.2%, see Table 2) were subject to paired-end sequencing on the Illumina Miseq. Raw sequences were quality-trimmed and mapped to a variety of databases, including a human database and a custom-made malaria database (see “Methods”), using MGmapper [18]. Table 2 lists the percentages of quality trimmed raw reads mapping to human, malaria and other databases. On average, the parasite content was 61% across the five samples, ranging however from 46 to 82%. In comparison, initial demonstration of leukocyte depletion with Plasmodipur filters revealed a median parasite content of 36.6% for samples ranging in parasitaemia from 0.7 to 9.9% [8], while demonstration of a comparable protocol applying CF11 cellulose resulted in an average parasite content of 66%, for samples with parasitaemias ranging from 0.4 to 7.3% [9]. Studies applying sWGA have demonstrated an average parasite content of 70% [10, 11]. The percentages of malaria, human and other reads in the samples analysed in the current study are not clearly correlated with parasitaemia of the infection, as has also been seen before [8]. The discrepancies may mainly be due to suboptimal leukocyte depletion in some samples (sample 1 contains 35% human reads) and/or the presence of environmental contamination of the sample, such as bacteria (sample 5 contains 36% “other”), as the samples are not processed under sterile conditions. The possibility of environmental contamination was anticipated, and illustrates the necessity of filtering the raw reads bioinformatically.

**Table 2:** Samples selected for WGS

Sample	Parasite count	Leukocyte count	Parasitaemia (paras./μl)	Parasitaemia (%)	Malaria (%)	Human (%)	Other (%)	Average read depth	Coverage
1	98	200	3920	0.1	46.1	35.2	18.7	16×	87.5
2	214	200	8560	0.2	67.1	13.7	19.2	15×	88.4
3	420	200	16,800	0.4	62.2	6.4	31.4	41×	95.3
4	500	102	39,216	1.0	81.7	3.5	14.8	67×	97.8
5	500	83	48,193	1.2	48.1	15.6	36.3	99×	98.9

Parasitaemia is given as parasites/µl (as described in Table 1, and in “Methods”) as well as in percentage, which is calculated according to an assumed erythrocyte count of 4000,000 erythrocytes per µl whole blood. Sequencing reads were mapped to a variety of databases, including a human database and a malaria database, using MGmapper (see “Methods”) [18]. The percentage of raw reads mapping to human, malaria and other databases are listed, as well as the average read depth of the sample and coverage as compared to the 3d7 reference genome

**Fig. 2:**
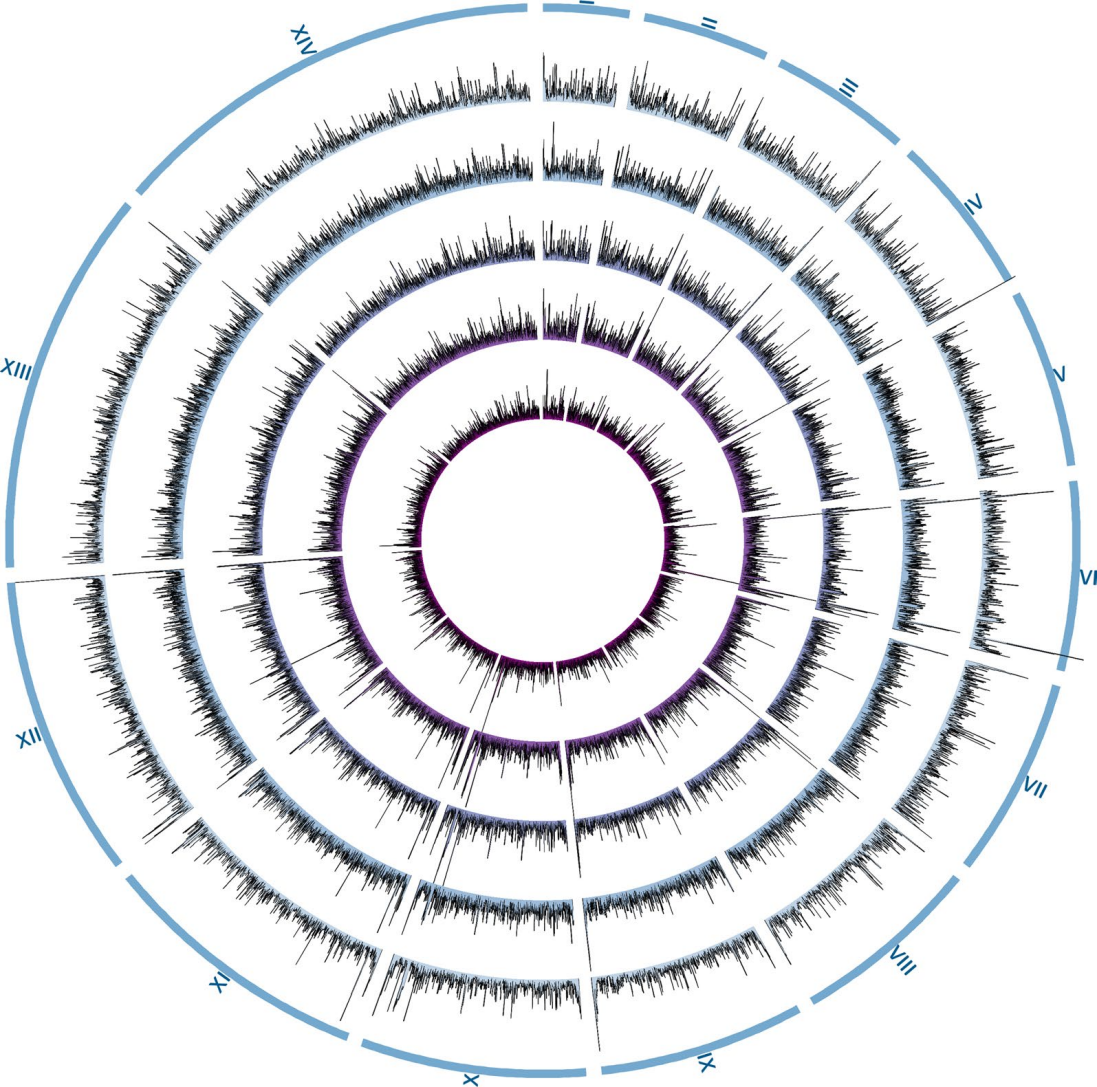
Read distribution across reference genome. From outermost ring: 3D7 reference genome chromosomes 1–14 (number written in roman letters, chromosomes illustrated to scale). Histograms representing read depths averaged over 2000 bp for sample 5, sample 4, sample 3, sample 2 and sample 1 (such that parasitaemia decreases from outer to inner most ring). The image was produced using the Circos software (see “Methods”) [22]

**Fig. 3:**
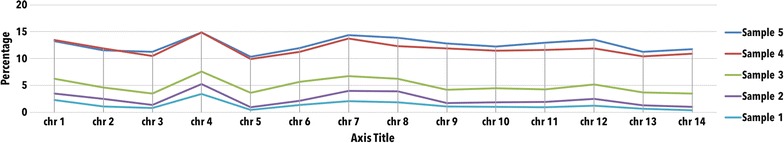
Chromosomal distribution of uncovered bases. The percentage of uncovered bases of each chromosome (number of uncovered bases on chromosome/size of chromosome *100) is depicted for each sample

Reads mapping to the malaria-database were aligned to the 3d7 reference genome, to assess the coverage and depth obtained for the individual samples (Table 2). The data clearly demonstrate the expected relationship between parasitaemia of the infection and resulting coverage and read depth, illustrating that lower parasitaemia infections will require more sequencing to attain the same depth of coverage. The sample with lowest parasitaemia (sample 1, corresponding to 0.1%) resulted in a coverage of 87.5% of the 3d7 reference genome, and an average read depth of 16× (≈ 370 million bp). These results are comparable to results obtained using sWGA [10], where an infection with 0.1% parasitaemia gave coverage of ~ 90% with 400 million bp sequenced (depth = 17.5×). The samples with highest parasitaemia (sample 4 and 5) averaged on a coverage of the 3d7 reference genome of 98.4% and a read depth of 83× (sample 4 (1% parasitaemia) = 67 × depth and 97.8% coverage and sample 5 (1,2% parasitaemia) = 99× depth and 98.9% coverage). Although similar read depths have not been demonstrated for sWGA studies on *P. falciparum*, the sWGA studies indicate that a 3d7 reference genome coverage above 90% is difficult to achieve at 1% parasitaemia [10], or solely the core genome coverage is assessed, also just surpassing 90% coverage [11], which is likely due to low amplification of certain regions in the genome. The overall distribution of read depth acquired in the current study, is depicted in Fig. 2 as circular diagrams representing each of the five samples across all 14 3d7 reference chromosomes. Together with the chromosomal-percentage of uncovered bases (percentage of chromosome size not covered, Fig. 3), the data indicate a relatively evenly distributed sequencing depth across the genome, including subtelomeric regions, with lower-parasitaemia samples capable of generating comparable data to higher-parasitaemia samples, given the extra sequencing capacity. Also for sWGA studies, it has been shown that lower-parasitaemia samples mimic higher-parasitaemia samples in read distribution across the genome [10, 11].

Relatively small “dents” in the read depths can be seen centrally on chromosomes 4 and 7 (Fig. 2), mirrored by peaks of uncovered bases for these chromosomes on Fig. 3. These areas correspond to clusters of *var* genes located in these chromosomes, and may be caused by difficulty in mapping to these highly variable regions, but may also be caused by difficulties in sequencing these regions due to their increased tendency to form secondary structures in general [24]. The same “dents” have been shown for sWGA protocols [10].

### SNP-analysis

The samples subject to whole-genome sequencing in this study have previously been subject to targeted sequencing [21], for analysis of resistance-conferring mutations in *pfmdr1*, *pfdhfr*, *pfcrt*, *pfdhps* and *pfk13*. The WGS data were, therefore, compared to the targeted sequencing data in order to assess the reliability of the WGS data. The results are listed (see Additional file 1: Table S1A), and the percentages of the genes covered by the WGS data are listed (see Additional file 1: Table S1B). All resistance-conferring SNP data for these five samples were confirmed. The only gene to not be covered 100% in the samples with lowest parasitaemia was *pfcrt*, which contains 12 AT-rich introns and, therefore, is expected to be more difficult to sequence, align and assemble. This not only confirms the reliability of the WGS data, but also illustrates that reliable SNP-analyses can be performed based on samples with coverage around 90% and an average read depth of 16×.

## Conclusion

This study shows that venous blood collected and processed without use of electricity, stored as dried erythrocyte spots at ambient temperatures in rural settings can be used for direct WGS of *P. falciparum*. Sampling for direct WGS was successful for infections with as few as 1000 parasites/µl. The method thus enables sampling for direct WGS, in areas where previously described protocols are unsuitable.

## Additional file

**Additional file 1: Table S1A.** SNP analysis was performed for all five samples for *pfdhfr*, *pfmdr1*, *pfcrt*, *pfdhps* and *pfk13*. The data confirmed previously performed SNP analysis for the samples, performed through targeted sequencing [21]. Grey fields indicate mutations found in the samples. **Table S1B.** Coverage of the genes analysed for polymorphisms in Additional file 1: Table S1A are listed for each sample.

## Abbreviations

DBS: dried blood spot
DES: dried erythrocyte spot
DNA: deoxyribonucleic acid
NGS: next-generation sequencing
SNP: single-nucleotide polymorphism
WGA: whole genome amplification
WGS: whole genome sequencing
