# Supplementary material for: Direct whole-genome sequencing of Plasmodium falciparum specimens from dried erythrocyte spots
Source: Malar J. 2018 Feb 23;17:91. doi: 10.1186/s12936-018-2232-6 (PMC5824530; doi:10.1186/s12936-018-2232-6)
Supplement: Supplementary file 1 — Additional file 1: Table S1A. SNP analysis was performed for all five samples for pfdhfr, pfmdr1, pfcrt, pfdhps and pfk13. The data confirmed previously performed SNP analysis for the samples, performed through targeted sequencing [21]. Grey fields indicate mutations found in the samples. Table S1B. Coverage of the genes analysed for polymorphisms in Additional file 1: Table S1A are listed for each sample. [file 12936_2018_2232_MOESM1_ESM.pdf]

Supplementary Table 1A. SNP analysis

gene	pfdhfr					pfmdr1					pfcrt					pfdhps						pfk13
codon	16	51	59	108	164	86	184	1034	1042	1246	72	73	74	75	76	431	436	437	540	581	613	189
3d7	A	N	C	S	I	N	Y	S	N	D	C	V	M	N	K	I	S	G	K	A	A	T
Sample 1	A	N	C	S	I	Y	F	S	N	D	C	V	I	E	T	I	S	A	K	A	A	T
Sample 2	A	I	R	N	I	N	F	S	N	D	C	V	I	E	T	I	S	G	K	A	A	N
Sample 3	A	N	C	S	I	N	Y	S	N	D	C	V	M	N	K	I	S	G	K	A	A	T
Sample 4	A	I	R	N	I	N	Y	S	N	D	C	V	I	E	T	I	S	G	K	A	A	T
Sample 5	A	I	R	N	I	N	Y	S	N	D	C	V	M	N	K	I	A	A	K	A	A	T

Supplementary Table 1B. Individual gene-coverage

	pfdhfr	pfmdr1	pfcrt	pfdhps	pfk13
Sample 1	100%	100%	100%	100%	100%
Sample 2	100%	100%	100%	100%	100%
Sample 3	100%	100%	100%	100%	100%
Sample 4	100%	100%	69%	100%	100%
Sample 5	100%	100%	79%	100%	100%
